# High Frequency of Diabetic Ketoacidosis in Children with Newly Diagnosed Type 1 Diabetes

**DOI:** 10.1155/2016/9582793

**Published:** 2015-12-13

**Authors:** Agnieszka Szypowska, Anna Ramotowska, Monika Grzechnik-Gryziak, Wojciech Szypowski, Anna Pasierb, Katarzyna Piechowiak

**Affiliations:** Department of Paediatrics, Medical University of Warsaw, Dzialdowska 1, 01-184 Warsaw, Poland

## Abstract

*Aim*. The aim of this study was to evaluate the incidence of diabetic ketoacidosis in children and adolescents with newly diagnosed type 1 diabetes in 2006-2007 and 2013-2014. *Method*. The study group consisted of 426 children aged 0–18 years with type 1 diabetes onset admitted to our hospital in 2006-2007 (group A) and 2013-2014 (group B). The study comprised the analysis of medical and laboratory records from patients' medical charts and the electronic database. *Results*. There was no difference between groups A and B in the percentage of children admitted with diabetic ketoacidosis (25% versus 28%, resp., *P* = 0.499). Among children with diabetic ketoacidosis, severe metabolic decompensation (pH < 7.1) appeared in similar frequency in groups A and B (28% versus 30%, resp., *P* = 0.110). In group B, children with diabetic ketoacidosis were statistically younger compared to patients without ketoacidosis (*P* = 0.015) and had higher HbA1c levels (*P* = 0.006). In both groups, a 2-fold increase in diabetic ketoacidosis was noted in children under the age of 3, compared to overall frequency. *Conclusion*. No decrease in diabetic ketoacidosis has been noted in the recent years. Although the prevalence and severity of diabetic ketoacidosis remain stable, they are unacceptably high. The youngest children are especially prone to ketoacidosis.

## 1. Introduction

The incidence rate of type 1 diabetes has increased worldwide, with the greatest rise in annual incidence among children under the age of five. The overall incidence rate in the region of Silesia in Poland rose 3.8 times, with the highest annual increment of the incidence rate being in children between 5 and 9 years of age [1].

Prolonged insulin deficiency in patients with newly recognized type 1 diabetes (T1D) may lead to diabetic ketoacidosis (DKA). Previous studies report worldwide variation in the frequency of DKA at diabetes onset ranging from 12.8% to 80% [2]. DKA is a potentially life-threatening acute complication due to cerebral oedema which is noted in 0.3–1% of newly diagnosed cases [3]. Recent studies highlight the fact that subtle cerebral injury might occur even when clinically apparent cerebral oedema is not observed [3]. Cameron et al. demonstrate alterations in cerebral white matter, particularly in the frontal lobes, which are most prominent in the youngest children with the most severe DKA. These brain changes are associated with persisting alterations in attention and memory [4]. Moreover, admission of a child with DKA increases the cost of diabetes treatment. The mean excess medical expenditure associated with one episode of DKA in privately insured U.S. children was calculated to be over $3.500 [5].

The incidence of DKA at diabetes onset may be due to parents' unawareness of symptoms of hyperglycaemia. Having a first degree relative with diabetes is associated with a decreased risk of DKA at diabetes. However, the degree of awareness of diabetes symptoms among medical care providers seems to be crucial in DKA prevention [6]. If diabetes is not diagnosed on the first visit to the physician, the risk of DKA increases significantly. Other factors such as insurance, parental education, distance to diabetes centre, and aggressive form of diabetes may also influence the risk of DKA in children with diabetes. Our previous analysis performed from January 2006 to March 2008 showed a 26% rate of DKA at the onset of diabetes [7]. Due to a rising incidence rate of type 1 diabetes in Polish children and greater awareness of diabetes, we expected a decrease in DKA at diagnosis. The aim of this study was to evaluate the incidence rate of DKA in children with newly diagnosed type 1 diabetes admitted to our centre in 2013 and 2014 in comparison with patients admitted five years earlier in 2006 and 2007.

## 2. Materials and Methods

The study group consisted of 426 children with newly diagnosed type 1 diabetes mellitus admitted to the Department of Paediatrics at the Medical University of Warsaw, Poland, between 2006 and 2007 (group A) and between 2013 and 2014 (group B). An additional analysis was performed in different age groups: below 5 years of age, 5–9 years of age, and over 9 years of age. The study comprised the analysis of medical and laboratory records from patients' medical charts and the electronic database.

Diabetes ketoacidosis was defined as blood glucose > 11 mmol/L and capillary pH < 7.3. Severity of ketoacidosis was categorized depending on the severity of acidosis: from severe with pH < 7.1 to moderate with pH < 7.2 and mild with pH < 7.3 [8].

Metabolic control was measured by glycated haemoglobin (HbA1c). In 2006-2007, HbA1c was measured using NGSP-certified Bio-Rad Variant with nondiabetic values ranging from 4.0 to 5.8%. HbA1c was calculated automatically with the Variant algorithm and the results were aligned with the Diabetes Control and Complication Trial. In 2014-2015, HbA1c was measured using high-performance liquid chromatography (reference range 4.0–6.0%, Bio-Rad Polska Tosoh 2.2; Tosoh Bioscience, South San Francisco, CA).

### 2.1. Statistical Analysis

The results are given as mean values with standard deviations (SD). The Gaussian distribution was tested using D'Agostino and Pearson omnibus normality test. The differences in outcome measures between groups were made using Student's *t*-test (unpaired, two-tailed), Mann-Whitney *U* statistic, Fisher's exact test, and the Tukey-Kramer multiple comparisons test. *P* values < 0.05 were considered statistically significant. The analysis was performed using StatsDirect v. 2.8.0 (England, StatsDirect Ltd., 2013).

## 3. Results

Children admitted to our hospital with newly diagnosed T1D in 2006-2007 (group A) and 2013-2014 (group B) had similar pH at diabetes recognition (Table 1). There was no difference between groups A and B in the percentage of subjects admitted with ketoacidosis (25% versus 28%, resp., OR 0.84 95% CI 0.54–1.32, *P* = 0.499). We did not note any differences between groups A and B regarding the severity of ketoacidosis. Among children with DKA in group A compared to group B, mild ketoacidosis (pH < 7.3–7.2≥) was observed in 52% (21/40) versus 43% (32/74) of cases, respectively, OR 1.45 95% CI 0.67–3.1, *P* = 0.432; moderate ketoacidosis (pH 7.2–7.1>) was noted in 20% (8/40) versus 27% (32/74) of cases, respectively, OR 0.68 95% CI 0.27–1.7, *P* = 0.497; and severe ketoacidosis (pH < 7.1) appeared in 28% (11/40) versus 30% (22/74) of cases, respectively, OR 0.50 95% CI 0.22–1.1, *P* = 0.110.

In group B, children with DKA were statistically younger compared to patients without DKA (8.0 ± 4.4 versus 9.5 ± 4.4, resp.; *P* = 0.015) and had statistically higher HbA1c values (12.4 ± 1.8 versus 11.7 ± 1.6, resp.; *P* = 0.006). The mean age of children in group A with DKA was lower than that of patients without DKA; however, without statistical difference (8.4 ± 5.0 versus 9.3 ± 4.4, resp., *P* = 0.314), there was no difference in HbA1c values between groups A and B (11.7 ± 1.2 versus 11.3 ± 2.4, resp., *P* = 0.236).

**Figure 1:**
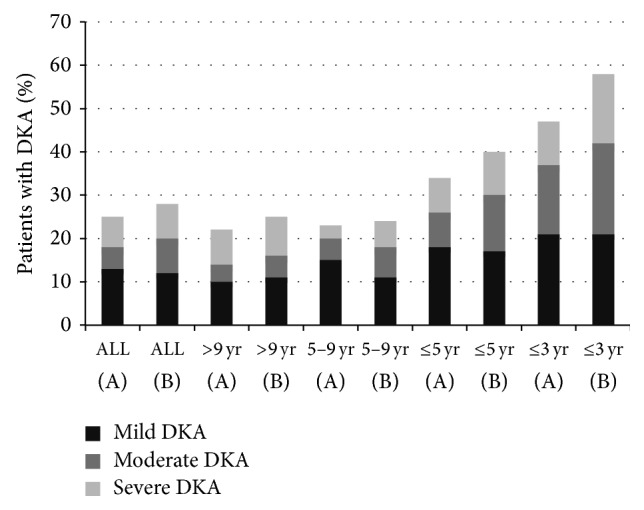
The rate of children with diabetic ketoacidosis (DKA) and distribution by severity of DKA in groups A (2006-2007 years) and B (2013-2014 years) in all patients and in different age groups.

There was no statistical difference in the prevalence of ketoacidosis in different age groups between groups A and B. In both groups, ketoacidosis occurred more frequently in children under the age of 5 than in older subjects (Table 2). However, there was no difference between groups A and B in the prevalence of DKA in children under the age of 5, *P* = 0.520. Moreover, the analysis of children under the age of 3 showed that there was no statistical difference in DKA between groups A and B; and pH < 7.3 was noted in 9/19 (47%) versus 14/24 (58%) of cases, respectively, OR 0.64 95% CI 0.19–2.16, *P* = 0.547. The comparison between age groups (<5 years, 5–9 years, and >9 years) showed the lowest pH in children under the age of 5 (*P* = 0.082, *P* = 0.028, resp.) in both groups A and B. In group A, patients younger than 5 years had the lowest HbA1c compared to patients between 5 and 9 years of age and over 9 years of age (*P* = 0.0002). In group B, HbA1c was similarly low in children under 5 years of age and between 5 and 9 years of age compared to older participants (*P* = 0.082). Among children with DKA, there was no difference between groups A and B in the incidence of mild and severe DKA (pH < 7.2) in different age groups. In group A compared to group B, mild and severe DKA was noted in children under 5 years of age in 6/32 (50%) versus 14/24 (58%) cases, respectively, OR 0.32 95% CI 0.10–1.08, *P* = 0.078; in children between 5 and 9 years of age in 3/9 (33%) versus 9/17 (53%) cases, respectively, OR 0.44 95% CI 0.08–2.39, *P* = 0.429; and in children over 9 years of age in 10/19 (53%) versus 19/33 (57%) cases, respectively, OR 0.82 95% CI 0.26–2.54, *P* = 0.778 (Figure 1).

In both groups A and B, no death occurred at diabetes recognition.

## 4. Discussion

Our study showed no decline in the frequency of DKA in children and adolescents with newly recognized type 1 diabetes admitted to our hospital between 2006 and 2007 and five years later between 2013 and 2014. Still, over one-quarter of patients between 0 and 18 years of age manifested with DKA at first diagnosis of diabetes. Previous analyses show that DKA was diagnosed in about 22–26% of Polish children with new onset of T1D [7, 9, 10]. An increasing prevalence of T1D has been noted in Polish children and some authors indicate that the frequency of DKA at T1D onset is inversely proportional to the baseline incidence of diabetes [11]. In countries with low prevalence of T1D, symptoms of diabetes might be less familiar to health care professionals, which might lead to misdiagnosis and increased occurrence of DKA at diabetes recognition. Our previous report and reports of other authors show that misdiagnosis at the initial visit to the doctor was associated with a higher rate of presentation with DKA [7]. We hypothesized that the awareness of symptoms of diabetes increased in both the general population and among the health care professionals in our district and we expected a decrease of DKA at diabetes onset. Unfortunately, we noted a slightly upwards trend in the frequency of DKA in our patients. Between 2006 and 2007, DKA was reported in 25% of children and between 2013 and 2014 DKA was noted in 28% of patients with newly recognized diabetes admitted to our hospital. The incidence of DKA at diagnosis of T1D in our children was similar to that in some countries such as UK (25%) [12], Germany (27%) [13], or USA (30%) [14] and lower than that, for example, in France (43.9%) [15], Austria (37.2%) [16], Brazil (42%) [17], or Nigeria (77%) [18]. Reported rates of DKA were higher in our study than in Sweden (16%) [2], Canada (18.6%) [19], and Finland (19.4%) [20].

There was no difference between both study periods 2006-2007 and 2013-2014 in the prevalence of ketoacidosis in different age groups (<5 years, 5–9 years, and >9 years). Ketoacidosis occurred more frequently in younger children. In 2013-2014, ketoacidosis was diagnosed in 40% compared to 32% of children under 5 years of age admitted to our hospital in 2006-2007. Our results are consistent with the results obtained by other authors. Similar incidence rate of DKA in children under 5 years of age was noted in UK (37%) [21], Austria (46%) [16], and France (44%) [15]. As expected, the highest prevalence of DKA was found in children younger than 3 years of age (58% in 2013-2014 and 47% in 2006-2007). Similar incidence of DKA in this age group was noted in USA (54%) [22] and lower one was noted in Canada (40%) [19].

The analysis showed a slightly upwards trend in the severity of DKA. In 2013-2014, mild and severe DKA (pH < 7.2) occurred in 57% of children with DKA at diabetes onset in comparison to 48% of patients in 2006-2007. In both groups, one-third of children with DKA had severe acidosis with pH < 7.1. In both time periods, the severity of DKA was similarly high in different age groups, except the youngest children under 3 years of age, who were more likely to present with moderate or severe DKA.

When compared to other centres (Teddy study: 11.3%, SEARCH study: 36.4%, German DPV register: 25.3%, Swediabkids: 16.9%, and Finnish register: 18.7%) [23], the number of mild and severe episodes of DKA in our population remains high. Moreover, children with DKA had higher HbA1c compared to youth without DKA, which may indicate longer duration of the preclinical disease state.

Our study showed that the prevalence and the severity of DKA diagnosed remain stable among youth with type 1 diabetes onset admitted to our hospital. Similarly, stable and high prevalence in DKA was noted by other authors [14, 16, 21]. Moreover, in some countries, high prevalence of DKA did not change despite the implementation of community-based information programs [16, 21]. On the other hand, some authors reported that the frequency of DKA significantly decreased [24] after education campaigns [25]. It is not clear why education programs caused a reduction in the frequency of DKA in some countries and not in others.

The limitation of our study is its one centre design. Our study was performed in one centre, where children from one Polish region (Mazowieckie district) were admitted. Unfortunately, there is no national registry of diabetes in Poland. Therefore, to evaluate the incidence of DKA in a representative group of Polish children, a multicenter study is needed.

## 5. Conclusions

No decline in DKA was noted in youth with newly type 1 diabetes admitted to our hospital between 2006 and 2007 and five years later between 2013 and 2014. Although the prevalence and severity of DKA remained stable, it was unacceptably high. More than one-quarter of T1D children manifested with DKA at first diagnosis of diabetes. About half of the children with DKA reported mild and severe DKA. The youngest children were more prone to DKA, and the frequency of DKA increased 2-fold in children under the age of 3 compared to the general population. A multicenter study is needed to evaluate the frequency and causes of DKA in Polish children with type 1 diabetes.

## Figures and Tables

**Table 1:** Comparison between children with newly diagnosed T1D in 2006-2007 (group A) and 2013-2014 (group B).

	Group A (2006-2007)	Group B (2013-2014)	*P*
Number of participants	162	264	—
Female/male	67/95	118/146	0.546
Age (years)	9.1 ± 4.6	9.1 ± 4.4	0.932
Number ≤5 yr (%)	37/125 (23)	60/206 (23)	0.100
Number <5–9≥ yr (%)	39/123 (24)	71/193 (27)	0.569
Number >9 yr (%)	86/76 (53)	133/131 (50)	0.618
BMI (kg/m^2^)	16.8 ± 3.2	16.9 ± 3.3	0.942
HbA1c (%)	11.4 ± 2.2	11.9 ± 2.4	0.047
pH	7.33 ± 0.13	7.32 ± 0.12	0.135
Number pH <7.3 (%)	40 (25)	74 (28)	0.499

BMI: body mass index, HbA1c: glycated haemoglobin.

**Table 2:** Comparisons between groups in regard to age.

	Age ≤5 years			Age <5–9≥ years			Age >9 years		
	Group A	Group B	*P* value	Group A	Group B	*P* value	Group A	Group B	*P* Value
Number of participants	37	60	—	39	71	—	86	133	—
Age (years)	2.9 ± 1.3	3.2 ± 1.2	0.205	6.9 ± 1.2	7.0 ± 1.1	0.882	12.7 ± 2.5	12.9 ± 2.4	0.457
HbA1c (%)	10.6 ± 1.6	11.2 ± 1.9	0.029	11.2 ± 1.8	11.8 ± 2.2	0.121	12.0 ± 2.4	12.2 ± 2.7	0.164
pH	7.30 ± 0.2	7.29 ± 0.1	0.699	7.35 ± 0.11	7.34 ± 0.12	0.291	7.33 ± 0.13	7.32 ± 0.13	0.203
pH <7.3 *n* (%)	12/37 (32)	24/60 (40)	0.520	9/39 (23)	17/71 (24)	0.100	19/86 (22)	33/133 (25)	0.746
